# A Methylotrophic Bacterium Growing with the Antidiabetic Drug Metformin as Its Sole Carbon, Nitrogen and Energy Source

**DOI:** 10.3390/microorganisms10112302

**Published:** 2022-11-21

**Authors:** Pauline Chaignaud, Christelle Gruffaz, Adrien Borreca, Stéphanie Fouteau, Lauriane Kuhn, Jérémy Masbou, Zoé Rouy, Philippe Hammann, Gwenaël Imfeld, David Roche, Stéphane Vuilleumier

**Affiliations:** 1 Génétique Moléculaire, Génomique, Microbiologie, UMR 7156 CNRS, Université de Strasbourg, 67000 Strasbourg, France; 2 Institut Terre et Environnement de Strasbourg, UMR 7063 CNRS, ENGEES, Université de Strasbourg, 67000 Strasbourg, France; 3 Génomique Métabolique, Genoscope, Institut de Biologie François Jacob, Commissariat à l’Energie Atomique (CEA), Centre National de la Recherche Scientifique (CNRS), Université d’Evry, Université Paris-Saclay, CEDEX, 91057 Evry, France; 4 Plateforme Protéomique Strasbourg-Esplanade, Institut de Biologie Moléculaire et Cellulaire, FR 1589 CNRS, CEDEX, 67084 Strasbourg, France

**Keywords:** metformin, dimethylamine, guanylurea, methylotrophy, *Aminobacter*, micropollutants, biodegradation, genome analysis, functional genomics, mini-transposon mutagenesis

## Abstract

Metformin is one of the most prescribed antidiabetic agents worldwide and is also considered for other therapeutic applications including cancer and endocrine disorders. It is largely unmetabolized by human enzymes and its presence in the environment has raised concern, with reported toxic effects on aquatic life and potentially also on humans. We report on the isolation and characterisation of strain MD1, an aerobic methylotrophic bacterium growing with metformin as its sole carbon, nitrogen and energy source. Strain MD1 degrades metformin into dimethylamine used for growth, and guanylurea as a side-product. Sequence analysis of its fully assembled genome showed its affiliation to *Aminobacter niigataensis*. Differential proteomics and transcriptomics, as well as mini-transposon mutagenesis of the strain, point to genes and proteins essential for growth with metformin and potentially associated with hydrolytic C-N cleavage of metformin or with cellular transport of metformin and guanylurea. The obtained results suggest the recent evolution of the growth-supporting capacity of strain MD1 to degrade metformin. Our results identify candidate proteins of the enzymatic system for metformin transformation in strain MD1 and will inform future research on the fate of metformin and its degradation products in the environment and in humans.

## 1. Introduction

Our planet has been exposed to an ever-increasing diversity of chemicals as the result of industrial activities, and in particular pharmaceuticals [1]. Dissemination of active pharmaceutical ingredients into the environment [1,2] may affect ecosystem functioning, and challenge the safe operating space of the planetary boundary for novel entities [3]. Through their prominent roles in biogeochemical cycling, microorganisms are on the frontline for dealing with new chemical compounds, for example by developing the capacity to enzymatically transform them [4], or to tolerate their toxic effects.

Understanding how microorganisms are able to transform chemical environmental contaminants and to use them as nutrients for growth has been a major theme of environmental microbiology for several decades. Much is now known about the microbial degradation of high-volume chemicals such as halogenated solvents. There is increasing interest in characterising microorganisms capable of transforming or mineralizing other classes of widespread environmental contaminants such as pharmaceuticals (see e.g., [5,6,7] for recent representative studies). Many of these compounds are poorly or only partially degradable and are found in the environment together with their transformation products at very low yet biologically active concentrations.

Pharmaceuticals often have complex molecular structures, with functional groups which are key to their biological effects. This contributes to explain both their toxicity and persistence in the environment. We reasoned that specific types of microbial metabolism may be of particular relevance to the biodegradation of specific functional groups. In particular, microbial C1 metabolism [8,9,10], by transforming molecules lacking carbon-carbon bonds for growth, could be instrumental in degrading many pharmaceuticals with carbon atoms exclusively linked to heteroatoms such as nitrogen, oxygen or sulfur. In this context, the antidiabetic drug metformin ([11], possessing four carbon atoms but without C-C bonds, may perhaps be described as the ultimate “C1 pharmaceutical”.

Metformin is a first-line drug against type II diabetes, and one of the most prescribed antidiabetic agents (150 million people worldwide [12]). It is also considered for other therapeutic applications including cancer and endocrine disorders [12,13]. However, its mechanisms of action are not yet well-characterized [13]. Metformin remains largely unmetabolized by human enzymes and at daily doses of 0.5–2.5 g per patient [13], it has become a dominant micropollutant in wastewater treatment plants and aquatic environments [2,14,15,16]. Its potential toxic effects are still debated, with reported detrimental effects in aquatic organisms, and potentially also in humans (e.g., [14,16,17,18]), contrasting with studies suggesting the absence of significant risks [19]. Many *in situ* and laboratory microcosm studies have demonstrated metformin transformation, with guanylurea as a major transformation product [5,20,21,22,23,24]. The bacterial strain *Pseudomonas mendocina* GU was recently reported to be able to use guanylurea as a nitrogen source for growth, and the enzymes required for this metabolism have been identified and characterized [25,26]. Another study on the degradation of diverse micropollutants reported the isolation of an *Aminobacter* strain growing with metformin as the carbon source from a chemostat community [5], with guanylurea as an end-product, but the strain was not further characterized. Very recently, the genome sequences of two bacterial strains capable of using metformin as a nitrogen source have become available [27]. To the best of our knowledge, however, no microorganism capable of using metformin as a carbon and nitrogen source for growth has yet been reported.

Here, we describe the isolation of strain MD1 from wastewater treatment plant sludge, an aerobic bacterium capable of using metformin as the sole carbon, nitrogen and energy source for growth. The genome of strain MD1 was sequenced, its metabolism of metformin was investigated by proteomics and transcriptomics, and mini-transposon mutagenesis was used to identify genes involved in growth with metformin.

## 2. Materials and Methods

### 2.1. Isolation and Characterisation of Strain MD1

Strain MD1 was isolated from activated sludge of the wastewater treatment of Eurométropole Strasbourg (Strasbourg, France; 1 million inhabitant-equivalents) collected in January 2012. A sample (40 mL) of fresh aerobic sludge was centrifuged (12,000 rpm, 15 min) and resuspended in ultrapure water (20 mL). A 50 µL aliquot was used to inoculate 20 mL of liquid M3 mineral medium [28] with 10 mM metformin as the sole carbon and nitrogen source, and incubated at 30 °C with 100 rpm shaking for 11 days. The resulting culture (OD_600_ = 0.16) was restreaked on solid medium of the same composition. Colonies obtained after 5 days at 30 °C were reinoculated into fresh liquid medium and cultured as above for 3 days (final OD_600_ = 0.35), and strain MD1 was isolated as a single colony from a 10^4^ dilution of this culture.

Total DNA from sludge samples, enrichment cultures and the isolated strain was obtained using the UltraClean Soil DNA kit (MoBio, Carlsbad, CA USA) and Wizard genomic DNA purification kit (Promega, Madison, WI USA), respectively, following the manufacturer’s instructions. DNA concentrations were determined with the Quant-IT^TM^ Picogreen kit (Invitrogen, Thermo Fisher Scientific, Waltham, MA USA). The 16S rRNA gene of strain MD1 was PCR-amplified (TGradient thermocycler, Biometra, Eurobio Scientific, Courtaboeuf, France) with 10 ng total DNA from strain MD1 in a 10 μL reaction using 0.8 μM primers for both 27f and 1492r [29], 0.4 mM dNTPs, and iProof Taq polymerase in GC buffer (Bio-Rad, Hercules, CA USA). The PCR program involved initial denaturation at 95 °C for 2 min, 35 cycles of 94 °C denaturation, 55 °C hybridisation and 72 °C extension (1 min each), and a final 2 min extension at 72 °C. The PCR product was purified with the Geneclean Turbo kit (MoBio) according to the manufacturer’s instructions and sequenced using universal primers 27f and 1492r by standard procedures on an ABI 3170 sequencer (Applied Biosystems, sequencing platform of UPR 2357 CNRS, Strasbourg, France).

Quantification of the *Aminobacter* taxon during enrichment of strain MD1 was performed by qPCR using the *Aminobacter*-specific primer pair Ab151f/Ab585r [30] and primer pair 1369f/1492r for total rRNA copy number [31]. Briefly, 10^−4^–10^2^ ng total DNA from activated sludge starting material and liquid enrichment cultures were added to a 20 μL PCR mix in a 96-microplate well containing each primer pair at 0.3 μM, 5 mM MgCl_2_, in SYBR Green mix with HotGoldStar DNA Polymerase (Eurogentec, Liège, Belgium) according to the manufacturer’s instructions. PCR reactions were performed on a GeneAmp 5700 SDS (Applied Biosystems, Thermo Fisher Scientific), with a program of 5 min denaturation at 95 °C followed by 40 cycles of 15 s denaturation at 95 °C, 30 s hybridation at 54 °C, and 30 s extension at 72 °C. The number of gene copies per ng of DNA were determined from the observed threshold cycle.

Biolog GEN III Microplate^TM^ (Biolog Inc., Hayward, USA) assays, analyses of respiratory quinones and fatty acids, and experimental DNA-DNA hybridization of strains MD1, *A. niigataensis* DSM 7050^T^ and *A. aganoensis* DSM 7051^T^ were carried out by the Identification Service of Leibniz-Institute (DSMZ, Braunschweig, Germany), from cultures grown in M3 medium. Cells were disrupted with a Constant Systems TS 0.75 KW (IUL Instruments, Königswinter, Germany) and DNA in the crude lysate purified by chromatography on hydroxylapatite as described [32]. DNA-DNA hybridization was carried out as described [33] with modifications as in [34], using a model Cary 100 Bio UV/VIS spectrophotometer equipped with a Peltier-thermostatted 6 × 6 multicell changer and a temperature controller with an in situ temperature probe (Varian).

### 2.2. Growth Experiments

*Aminobacter* strain MD1, *A. niigataensis* DSM 7050^T^, *A. aganoensis* DSM 7051^T^ and strain Root100 [35] (obtained from the Deutsche Sammlung von Mikroorganismen und Zellkulturen GmbH (DSMZ, Braunschweig, Germany)), and strain MSH1 [36] (obtained from the Pasteur Institute Collection, France), were cultivated aerobically at 30 °C on solid or in liquid M3 mineral medium. Carbon and nitrogen substrates were added from stock solutions sterilized by filtration (0.22 mm, Sarstedt, Nümbrecht, Germany) after autoclaving of the medium. Metformin (1–2.5 mM), dimethylamine (2.5–5 mM) and sucrose (1–4 mM) were routinely used as carbon sources. Nitrogen-free M3 medium was used to evaluate ammonium sulfate (1.5 mM), methylamine (1.5 mM), dimethylamine (1.5 mM), trimethylamine (1.5 mM), metformin (1 mM) and guanylurea (1 mM) as potential nitrogen sources for growth. Antibiotics were used at final concentrations of 10 µg·mL^−1^ (tetracycline) and 30 µg·mL^−1^ (gentamycine) as required. Liquid cultures were grown in 20 mL medium in 100 mL Erlenmeyer flasks with agitation (100 rpm), and growth was monitored by measuring optical density at 600 nm (OD_600_). Comparison of growth rates with different carbon sources was performed in 96-well plates in a Xenius thermostatted microplate reader (safas monaco, Monaco, Monaco). Cultures were grown at 30 °C with 100 rpm shaking in 150 µL M3 mineral medium with either metformin (5 mM), dimethylamine (5 mM) or sucrose (1 mM) as the carbon source, starting from an exponential phase preculture diluted to a final OD_600_ of 0.01. Optical density was monitored automatically at 30 min intervals. Reported data represent the average of biological duplicate experiments performed in technical triplicates.

### 2.3. Analysis of Metformin and Transformation Products

Metformin utilization during growth was analysed from supernatants of bacterial cultures obtained following centrifugation of bacteria at 10,000 rpm for 10 min (MultifugeX1R, Heraeus, Hanau, Germany) at 4 °C. Metformin transformation was routinely followed by a Sakaguchi-type assay [37] adapted to a 96-well microtiter plate format (Sarstedt). Briefly, 70 µL of culture supernatant was mixed with 10 µL Sakagushi solution I (0.1% 1-naphthol; 1 N NaOH) and 20 µL freshly prepared Sakagushi solution II (0.3% active chlorine; 0.1 N NaOH). Absorbance was measured at 482 nm, 539 nm and 500 nm after 5 min incubation at room temperature using a microplate reader (BioTek, Agilent, Santa Clara, CA USA) and KC4™ software. Absorbance ratios 482/539 nm and 560/539 nm were used to estimate the formation of guanylurea and the degradation of metformin, respectively, based on the spectra of Sakaguchi adducts of reference samples of metformin and guanylurea (λ_max_ 482 nm) (Supplementary Figure S1). For quantitative measurements, metformin and its transformation products were quantified by ultra-high performance liquid chromatography (UHPLC, Ultimate 3000, Thermo Fisher Scientific) with an Accucore aQ C18 column (100 × 2.1 mm, 2.6 µm granulometry, Thermo Fischer Scientific), coupled to a triple quadrupole mass spectrometer (MS/MS, TSQ Quantiva, Thermo Fisher Scientific). Column and autosampler temperatures were 20 °C and 9 °C, respectively. Aqueous samples (10 µL) containing metformin-d6 internal standard at 200 µg L^−1^ (Sigma Aldrich, Merck, Darmstadt, Germany) were injected using an ACC-3000 autosampler (Ultimate 3000, Thermo Fisher Scientific). LC-grade water and acetonitrile, both acidified with 0.1% and 0.05% formic acid, respectively, were used as eluents at a flow rate of 0.3 mL/min with the following gradient: 10–40% acetonitrile (1 min), 40–90% acetonitrile (20 s), 90% acetonitrile (40 s), 90–10% acetonitrile (15 s), and 10% acetonitrile (30 s). The MS/MS spectrometer was operated at an ionization voltage of 1500 V in positive mode using collision-induced dissociation (CID) at 1.5 mTorr and a vaporizing temperature of 300 °C. Precursor and fragment ions were acquired in multi-reaction mode (MRM) and metformin and potential metabolites detected and quantified using reference standards (see Supplementary Table S1 for details). Metformin-d6 (Sigma-Aldrich) was used as an internal standard in all measurements.

### 2.4. Genome Sequencing, Assembly, Annotation and Analysis

Total DNA from strain MD1 was obtained from a 100 mL exponential culture grown with metformin at 30 °C in M3 medium, using the Masterpure DNA and RNA purification kit (Epicentre, Madison, WI USA) according to the manufacturer’s instructions. DNA sequencing was performed using a combination of Illumina Hiseq2500 and PacBio RS technologies (Baseclear, Leiden, The Netherlands). FASTQ sequence files from Illumina paired-end sequence reads were generated using the Illumina Casava pipeline version 1.8.3 (Illumina, San Diego, CA USA). The initial quality assessment was based on data passing Illumina Chastity filtering. Reads containing adapters and/or a PhiX control signal were removed using an in-house filtering protocol. The second quality assessment was performed on the remaining reads using the FASTQC quality control tool version 0.10.0 [38]. Data collected from the PacBio RS instrument were processed and filtered using the SMRT Analysis software suite (PacBio, Menlo Park, CA USA). Continuous Long Read (CLR) data were filtered by Read-length (>50), Subread-length (>50) and Read quality (>0.75). A draft assembly was performed using ABySS v1.5.1 [39] with the Illumina sequences trimmed off of low-quality bases (bbduk, part of BBMap v34.46) [40]. Scaffolding based on the alignment of the PacBio CLR reads and gap closure steps were performed using SSPACE-LongRead scaffolder v1.0 [41] and GapFiller v1.10 [42], respectively. The final assembly allowed for the definition of the chromosome and plasmid paAMD1 as circular objects. An alternative hybrid assembly launched with Unicycler v0.4.6 (default options) [43] allowed for the recovering of additional small plasmids pbAMD1 and pcAMD1 as circular objects. The automatic annotation of the genome was performed with the MicroScope platform pipeline (https://mage.genoscope.cns.fr/microscope accessed on 14 January 2022) [44]. Mean coverage depth for each replicon was computed using SAMtools [45]. For this, Illumina reads were first mapped against the assembled chromosome and the three plasmids using the alignment algorithm BWA-MEM of the BWA software package [46]. The MicroScope platform was also used for various bioinformatic analyses, notably for genome clustering (https://microscope.readthedocs.io/en/3.16.0/content/compgenomics/genoclust.html accessed on 14 January 2022), and for detection of gene homologs in related genomes of interest. In silico DNA-DNA hybridization analyses were performed using the genome-to-genome distance calculator (https://ggdc.dsmz.de accessed on 14 January 2022) [47] and JSpeciesWS (http://jspecies.ribohost.com/jspeciesws accessed on 14 January 2022) [48].

### 2.5. Proteomic Analysis

Strain MD1 was grown in triplicate 50 mL cultures at 30 °C with shaking, in M3 medium with metformin, dimethylamine or sucrose as the sole carbon source (10 mM total carbon). Cultures were harvested in the exponential phase (OD_600_ 0.14–0.17) and centrifuged (10 min, 8000 rpm, Heraeus MultifugeX1R). Cell pellets were first lysed in a final volume of 100 µL in an ice-water sonication bath with 1% Triton, 50 mM Tris-HCl pH 8.0, and 50 mM NaCl. After clarification by centrifugation (12,000 g, 4 °C, 15 min), the protein content in supernatants was quantified using a Bradford-based assay (Bio-Rad) using bovine serum albumin (BSA) as a reference.

Protein extracts were prepared as described previously [49]. Briefly, 10 µg of each sample were precipitated with cold 0.1 M ammonium acetate in 100% methanol, and proteins were resuspended in 50 mM ammonium bicarbonate. After reduction (5 mM dithiothreitol, 95 °C, 10 min) and alkylation (10 mM iodoacetamide, 20 min, room temperature), proteins were digested overnight with 200 ng of sequencing-grade porcine trypsin (Promega). The resulting vacuum-dried peptides were resuspended in water containing 0.1% (v/v) formic acid (solvent A). Peptide mixtures were analyzed using an Easy-nanoLC-1000 system coupled to a Q-Exactive Plus mass spectrometer (Thermo-Fisher Scientific) operating in positive mode with a nanoelectrospray source. A total of 750 ng of each sample were loaded on a C-18 precolumn (75 μm ID × 20 mm nanoViper, 3µm Acclaim PepMap; Thermo-Fisher Scientific) at 800 bars in solvent A. After desalting, the pre-column was switched online with the analytical C18 analytical column (75 μm ID × 25 cm nanoViper, 3µm Acclaim PepMap; Thermo-Fisher Scientific) and equilibrated in solvent A with 5% (v/v) solvent B (0.1% formic acid in acetonitrile). Peptides were eluted at a flow rate of 300 nL/min using a gradient from 5% B to 20% B in 120 min, from 20% B to 32% B in 15 min, from 32% B to 95% B in 1 min and 95% B to 95% B in 24 min. The Q-Exactive Plus was operated in data-dependent acquisition mode (DDA) with Xcalibur software (Thermo Fisher Scientific). Survey MS scans were acquired at a resolution of 70 K at 200 m/z (mass range 350–1250), with a maximum injection time of 100 ms and an automatic gain control (AGC) set at 3 × 10^6^. Up to 10 of the most intense multiply charged ions (≥2) were selected for HCD fragmentation with a normalized collision energy set at 27 eV, at 17.5 K resolution, with a maximum injection time of 100 ms and AGC set at 1 × 10^3^. A dynamic exclusion time of 20 s was applied during the peak selection process.

MS datasets generated by the mass spectrometer were searched against a homemade database for the strain MD1 database with a decoy strategy (5502 protein forward sequences) using the Mascot algorithm (version 2.5.1, Matrix Science, London, UK). The resulting dat Mascot files were then imported into Proline v1.4 package [50] for further post-processing. Proteins were validated on Mascot pretty rank equal to 1, 1% FDR on both peptide spectrum matches (PSM) and protein sets (based on Mascot score). Raw Spectral Count values were then imported into R (v3.2.5) in addition to the MSnbase and msmsTests libraries. The number of spectra were first transformed with the colSums function, affording column-wise normalization of the data matrix using spectral count values from all three replicates of each condition. Multidimensional scaling generated with the cmdscale() function allowed for the assessing of the similarity between replicates and conditions. Spectral count values were submitted to a negative-binomial test by edgeR GLM regression, and EdgeR results generated by the msms.edgeR() function were exported into Excel. Protein fold-change (FC) and adjusted *p*-value (adjp) corrected by Benjamini–Hochberg were computed for each identified protein. The threshold for differential protein production between 2 growth conditions was set at FC ≥ 2.0 with an adjusted *p*-value < 0.05. The R script used to process the dataset is available on Github (https://github.com/hzuber67/IPinquiry4 accessed on 14 January 2022).

### 2.6. RNA-Seq Analysis

Strain MD1 was grown in triplicate 150 mL cultures at 30 °C with shaking, in M3 medium with metformin, dimethylamine or sucrose as the sole carbon source (10 mM total carbon). Cultures were harvested in exponential phase (OD_600_ 0.15–0.17 for metformin and dimethylamine cultures, and OD_600_ 0.2–0.22 for sucrose cultures), and centrifuged (5 min, 10,000 rpm, Heraeus Multifuge X1R). Cell pellets were flash-frozen in liquid nitrogen and stored at −80 °C. Extraction, preparation and RNA sequencing was performed by Vertis Biotechnologie AG (Freising, Germany). Libraries were constructed in a strand specific manner as described previously [51]. Briefly, total RNA was isolated from the pellets using a bead mill and the mirVanaRNA isolation kit (Ambion, Thermo Fischer Scientific) including DNase treatment. Preparations were checked by capillary electrophoresis (MultiNA microchip electrophoresis system, Shimadzu, Noisel, Grance). Ribosomal RNA was depleted using the Ribo-Zero rRNA Removal Kit for bacteria (Illumina). The rRNA depleted RNA samples were first fragmented using ultrasonication (four pulses of 30 s each at 4 °C). An oligonucleotide adapter was then ligated to the 3’ end of the RNA molecules. First-strand cDNA synthesis was performed using M-MLV reverse transcriptase and the 3′ adapter as primer. The first-strand cDNA was purified and the 5’ Illumina TruSeq sequencing adapter was ligated to the 3’ end of the antisense cDNA. As a result, the following adapter sequences flank the obtained cDNA inserts: TruSeq_Sense_prime Barcode (5’-AATGATACGGCGACCACCGAGATCTACAC-NNNNNNNN-ACACTCTTTCCCTACACGA-GCTCTTCCGATCT-3′) and TruSeq_Antisense_prime (5′-CAAGCAGAAGACGGCATACGAGATGTGACTGGAGTTCAGACGTGTGCTCTTCCGATCT-3′) with a total combined length of the flanking sequences of 128 bases. Resulting cDNAs were PCR-amplified to about 10–20 ng/μL using a high-fidelity DNA polymerase and purified using the Agencourt AMPure XP kit (Beckman Coulter Genomics, Danvers, MA USA). After cDNA purification using the Agencourt AMPure XP kit (Beckman Coulter Genomics) and quality check using capillary electrophoresis, the obtained cDNA were pooled in approximately equimolar amounts and fractionated in the 200–500 bp size range using a differential clean-up with the Agencourt AMPure kit. Libraries were then sequenced on an Illumina NextSeq 500 system using 75 bp read length with a MID150 sequencing kit (Illumina). RNA-Seq sequence data were analysed using the RNA-Seq pipeline of the MicroScope platform [16]. In a first step, raw reads were mapped onto the strain MD1 reference genome using BWA-MEM v.0.7.4 [18]. An alignment score corresponding to at least half of the read was required for a hit to be considered. SAMtools (v.0.1.12) [22] was then used to extract reliable alignments with a Mapping Quality (MAPQ) ≥ 1 from SAM formatted files and to lower the false positive discovery rate. The number of reads matching each genomic object present on the reference genome was then computed the coverageBed tool of the BEDTools suite(v.2.10.1) [23]. Finally, the R package DESeq2 (v.1.22.2) [24] was used with lfcShrink function and its default parameters to normalize raw count data and test for differential expression between chosen growth conditions (metformin vs sucrose, dimethylamine vs sucrose, and metformin vs dimethylamine). For each comparison, differential gene expression was considered significant for a computed adjusted *p*-value ≤ 0.05 and a fold-change value ≥ 2.

### 2.7. Mini-Transposon Mutagenesis

A library of Gm^R^ promoterless gfp-based mini-transposon random insertion mutants of strain MD1 was constructed as described previously [52] in M3 medium with 2 mM sucrose as carbon source and gentamycin (30 μg/mL) for selection. Mutants obtained on M3 solid medium were picked manually, grown in the same liquid medium in 96-well microtiter plates, and stored at −80 °C. For screening, all obtained mini-transposon mutants were spotted onto solid M3 mineral medium plates prepared with Noble agar and containing 30 μg/mL gentamycin and 2 mM sucrose using a ROTOR robot (Singer Instruments, Roadwater, UK) and incubated for up to 4 days at 30 °C. Mutants were then transferred from these plates onto M3 mineral minimum medium plates containing 30 μg/mL gentamycin and 5 mM metformin or dimethylamine. Insertion sites of mini-transposon insertion were identified using a two-step PCR method and corresponding primers as previously described [52]. Amplicons were mapped to the MD1 genome sequence using the MAGE platform to determine mini-transposon insertion sites.

### 2.8. Data

The genome sequence data and RNA-Seq raw data were deposited in the European Nucleotide Archive (ENA) at EMBL-EBI within BioProject PRJEB52869. Chromosome and plasmid sequences have been deposited under the assembly accession numbers GCA_946995915 and OX341517-OX341520 for the chromosome and the 3 plasmids paAMD1-pcAMD1, respectively, and sample accession numbers ERS13482698 for Pacbio raw reads and ERS13638640 for Illumina raw reads, respectively. The genome sequence and its annotation are also accessible via the MicroScope platform. RNA-Seq raw data were deposited under sample accession numbers ERS13638631-ERS13638633 for the three replicates of the metformin growth condition, ERS13638634-ERS13638636 for the three replicates of the dimethylamine growth condition, and ERS13638637-ERS13638639 for the three replicates of the sucrose growth condition, respectively. RNA-Seq analysis results are available on the MicroScope platform [44] under RNAseq project RNAseq_B4KT2B_Aminobacter_MD1. Mass spectrometry proteomics data were submitted to the ProteomeXchange Consortium via the PRIDE partner repository under dataset identifier PXD036621 and 10.6019/PXD036621 for the genome-derived proteomics analysis. 

## 3. Results

### 3.1. Isolation and Characterization of Strain MD1

Aerobic enrichment cultures were set up with freshly collected aerobic sludge from the wastewater treatment plant of the city of Strasbourg as an inoculum, in the mineral medium routinely used in the laboratory [28], and with metformin as the sole carbon and nitrogen source. Growth was rapidly observed without lag, and isolates growing with metformin were readily obtained as colonies on agar plates of the same medium after only one liquid transfer. One purified colony was chosen for further characterization. The strain, identified as strain MD1, was shown to grow with methylated amines (methylamine, dimethylamine, trimethylamine, choline, creatinine), sugars, but not with methanol or succinate as the carbon source, as described previously for *Aminobacter* strains [53]. Beyond metformin, strain MD1 growing with sucrose could also use ammonium sulfate and methylated amines but not guanylurea as nitrogen sources for growth. In line with these growth characteristics, its 16S rRNA gene proved identical to that of *Aminobacter niigataensis* and *A. aganoensis* [54], thereby confirming its facultative methylotrophic metabolism. Analysis of enrichment cultures by qPCR using primers specific for the *Aminobacter* genus showed that *Aminobacter* was below the detection limit (<0.01% of total 16S rRNA sequences) in aerobic sludge used as inoculum, and was rapidly enriched (e.g., 2% of total 16S rRNA sequences after 10 days) under the chosen cultivation conditions with metformin as the sole carbon and nitrogen source.

Further physiological characterisation of strain MD1 confirmed typical characteristics of these *Aminobacter* type strains in terms of metabolism (Supplementary Table S1) [55]. Moreover, the fatty acid composition of strain MD1 was similar to that reported for closely related *Aminobacter* strains (Supplementary Table S2), and strain MD1 contains Q10 as its major quinone, as with other strains of the *Aminobacter* genus [54].

**Figure 1:**
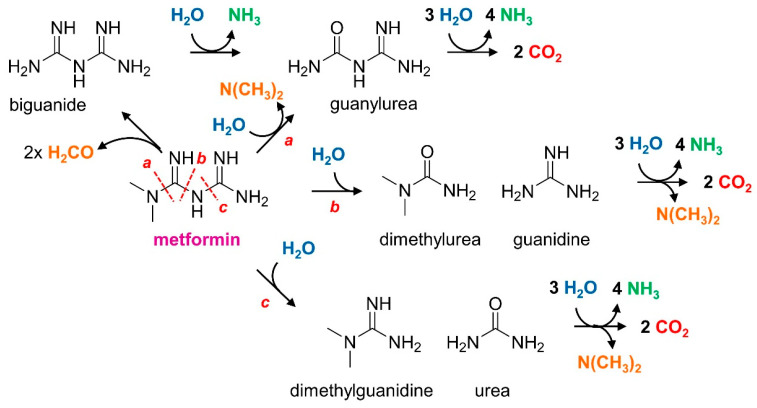
Potential pathways for the utilization of metformin as the sole carbon source for bacterial growth. Energy for growth from carbon is only available from the oxidation of the two methyl groups of dimethylamine (orange). Monooxygenases acting on these N-methyl groups will produce formaldehyde (left), which may then be oxidised to CO_2_, generating reducing equivalents for growth. Alternatively, hydrolysis of the two guanidine groups by carbon-nitrogen hydrolases (alternative pathways *a*–*c*) will release dimethylamine either directly (*a*) or at later stages of metformin degradation (*b*,*c*), with concomitant production of different intermediates with guanidine and/or amide functional groups, including but not limited to those shown. Energy for growth may also be derived from oxidation of ammonia (green) released by hydrolysis reactions.

Several pathways for growth supporting degradation of metformin were envisaged, most featuring formation of dimethylamine (Figure 1). The efficient growth of the strain with dimethylamine suggested that metformin degradation proceeded by hydrolysis of metformin rather than oxidation of the N-methyl groups to formaldehyde (Figure 1). Strain MD1, however, was unable to grow with guanylurea, or with other potential degradation intermediates such as dimethylguanidine, guanidine, dimethylurea, and urea as the carbon source (Figure 1).

**Figure 2:**
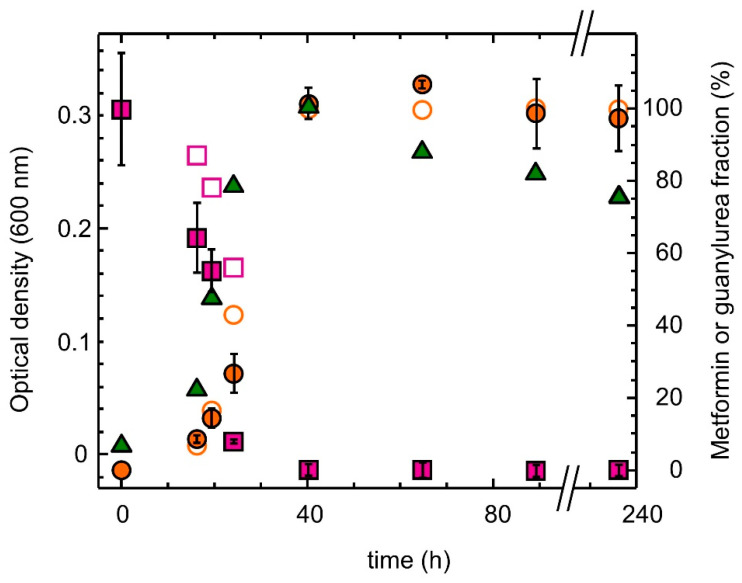
Transformation of metformin by strain MD1 growing in mineral M3 medium with metformin as the sole carbon, nitrogen and energy source. Metformin (purple squares) and guanylurea (orange circles) were quantified by LC/MS-MS analysis (full symbols) and also estimated by colorimetric analysis with the Sakaguchi reagent by normalization using reference compounds of known concentration analysed in parallel (open symbols). The growth of strain MD1 was followed by optical density at 600 nm (green triangles).

Quantitative analysis by LC/MS-MS of culture supernatants of strain MD1 grown with metformin demonstrated the formation of stoichiometric amounts of guanylurea upon the complete depletion of metformin in the medium (Figure 2, Supplementary Table S3). This confirmed the colorimetric estimation of metformin utilisation based on the Sakaguchi reaction [37] routinely used to monitor metformin transformation by strain MD1 (Figure S1). These results also provided further indication that strain MD1 uses the dimethylamine released from the hydrolysis of metformin for methylotrophic growth, leaving guanylurea as a side product.

### 3.2. Genome Analysis

The genome sequence of strain MD1 was obtained in fully assembled form by a combined Pacbio and Illumina sequencing strategy. It consists of a circular 4.97 Mb chromosome, a large circularised plasmid paAMD1 (363.6 kb), and two small circular plasmids (pbAMD1 and pcAMD1) of 24.9 and 21.6 kb, respectively (Table 1). Sequence coverage analysis from Illumina sequence data suggested that all three plasmids were single copy.

**Figure 3:**
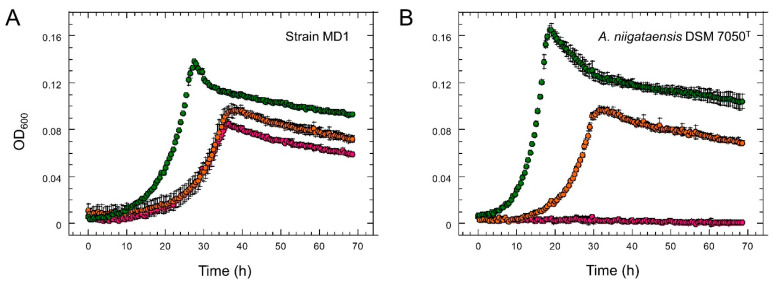
Growth of strain MD1 and *Aminobacter niigataensis* type strain with different carbon sources. Strain MD1 (**A**) and *A. niigataensis* type strain DSM 7050^T^ (**B**) were grown in mineral medium with metformin (5 mM, purple), dimethylamine (5 mM, orange) or sucrose (1 mM, green) as the sole carbon source. Curves represent the average of three replicate cultures, with error bars shown at one standard deviation.

The similarity of the genome sequence of strain MD1 to other *Aminobacter* strains [53] was evaluated by the MicroScope genome clustering tool [44], suggesting that strain MD1 was most closely related to the *A. niigataensis* type strain and to strain MSH1 (Figure S2). An in silico hybridization analysis of the genome sequence confirmed that strain MD1 belongs to the *A. niigataensis* species, as also suggested by experimental DNA-DNA hybridization (Supplementary Table S4). Most importantly, *Aminobacter* type strain *A. niigataensis* DSM 7050^T^, *Aminobacter* sp. strain MSH1 (recently assigned to the species *A. niigataensis* [36]), the *A. aganoensis* type strain DSM 7051^T^, and *Aminobacter* sp. strain Root100 which are all closely related to strain MD1 (Figure S2) did not grow with metformin as the sole carbon source (Figure 3).

The genome sequence of strain MD1 was annotated automatically using the MicroScope pipeline [44]. Genes potentially associated with transformation of metformin to guanylurea were searched based on functional annotation in EGGNOG and Interpro (Table 1), using the terms carbon-nitrogen hydrolase, ureohydrolase, amidohydrolase, and amidase. The term carboxylase was also searched, since enzymatic degradation of the guanidine function of metformin may involve a carboxylated intermediate, as reported for the hydrolysis of guanylurea [25,26]. Hence, based on sequence analysis alone a large number of gene candidates were potentially associated with the hydrolysis of metformin to guanylurea.

**Figure 4:**
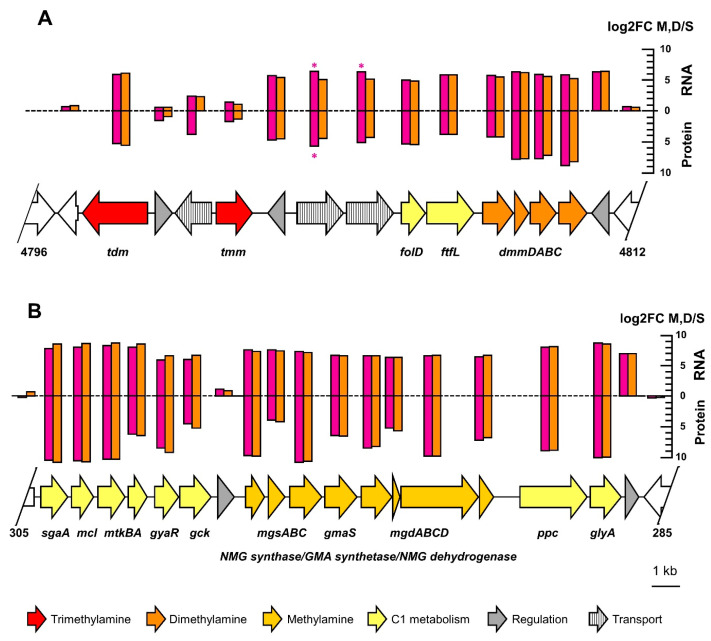
Genome regions associated with dimethylamine and methylamine utilization. CDS are colored according to the function predicted by automatic annotation as indicated. Significantly higher differential abundance of most CDS of gene clusters for utilization of dimethylamine (**A**) and methylamine (**B**) were observed in cultures grown with metformin (purple) or dimethylamine (orange), when compared to cultures grown with sucrose. However, only two CDS showed significant differences (*p* < 0.05) between growth with metformin or with dimethylamine relative to growth with sucrose (purple asterisks).

Genes for dimethylamine and trimethylamine utilization were identified on the chromosome and were associated with other genes of methylotrophic metabolism as observed previously ([58,59]; also see below, Figure 4A). In contrast, genes for methylamine utilization by the N-methylglutamate pathway [28,60] were detected on the large 363 kb plasmid paAMD1 with upstream and downstream gene clusters involved in methylotrophic metabolism (see below, Figure 4B). The plasmid localization of genes essential for methylotrophic metabolism, while somewhat surprising, is not unheard of [58]. No other genes for methylamine utilization, e.g., by PQQ-dependent methylamine dehydrogenase [61], were detected.

### 3.3. Comparative Proteomics and Transcriptomics

Global patterns of gene expression (Supplementary Table S5) and protein production (Supplementary Table S6) were investigated for growth of strain MD1 with metformin, dimethylamine and sucrose (Figure 3, Table 2). The three chosen growth substrates allowed for the identification of genes (i) specifically involved in metformin utilization, and (ii) involved in methylotrophic metabolism from dimethylamine released from metformin. The percentage of differentially abundant CDS relative to the total detected in RNA-Seq and proteomics experiments was very similar (Table 2). We focused on proteins and transcripts with higher relative abundance during growth with metformin (Table 2), which were considered to be more likely to be involved in metformin utilization.

Strikingly, most CDS showing higher production in the metformin growth condition than in the sucrose condition were also found in the dimethylamine versus sucrose comparison (Table 2). This further confirmed that strain MD1 grows methylotrophically by using dimethylamine formed upon hydrolysis of metformin. Specifically, transcripts and protein products of chromosomal genes for dimethylamine utilization (Figure 4A), as well as of plasmid-encoded genes for methylamine utilization (Figure 4B), showed large and similar changes upon growth with metformin or dimethylamine as compared to growth with sucrose. Indeed, only two CDS in these gene regions showed significant differences in abundance (*p* < 0.05) between growth with metformin and dimethylamine relative to growth with sucrose.

Similarly, relatively few transcripts and proteins were detected with significantly higher abundance in the metformin condition compared to both dimethylamine and sucrose (Table 2), and all of them were detected at low abundance. Finally, proteins detected only in the metformin condition all showed extremely low abundance (i.e., 1–2 spectral counts in total over the three replicate experiments, see Supplementary Table S6), tentatively suggesting that they were not involved in growth with metformin.

### 3.4. Mini-Transposon Mutagenesis

Mini-transposon mutagenesis of strain MD1 was performed to identify genes that were essential for metformin utilization, an approach we had previously used to discover essential genes for growth with chloromethane [62] and dichloromethane [52] in aerobic methylotrophic organisms of the Alphaproteobacterial genus *Methylobacterium*. To our knowledge, however, this genetic tool had not been previously applied to an *Aminobacter* strain. The suicide plasmid pAG408 carrying a mini-Tn5 transposon [63] was introduced into strain MD1 by conjugation, and gentamycin-resistant mutants of strain MD1 were selected on mineral medium M3 with sucrose as the carbon source. An initial Southern blot analysis of a few randomly picked mutants did not provide evidence of mutational hot spots for mini-transposon insertion. Single insertions of the mini-transposon and the stability of insertion were expected from previous studies [52,62,63] and were subsequently verified by Southern blot analysis and PCR analysis as required. Screening of the total 6780 mutants obtained yielded 20 mutants that did not grow with metformin (Supplementary Table S7). Among those, nine mutants were specifically unable to grow with metformin but grew with dimethylamine (Table 3). The precise mini-transposon insertion site in these mutants (Supplementary Table S7) was identified as described previously [52]. All insertion sites were different as expected for the random nature of transposon Tn*5*-based insertions. Functional annotation of the corresponding genes suggested potential roles in the hydrolysis of C-N bonds, as well as in transport and regulatory processes (Table 3).

**Figure 5:**
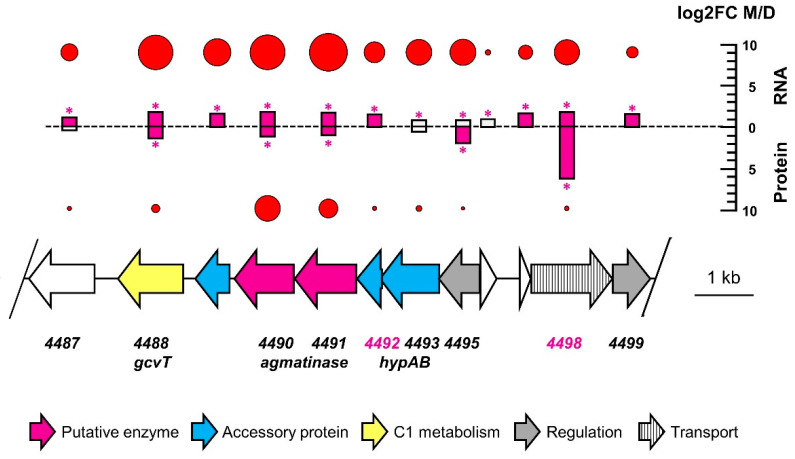
Chromosomal 4488–4498 gene cluster of strain MD1. Gene synteny in this genome region actually extends from gene 4472 to gene 4628 for *Aminobacter niigataensis* strains MD1, MSH1 and the type strain DSM 7050^T^. Genes inactivated by mini-transposon in strain MD1 mutants specifically unable to grow with metformin are indicated in purple. Log_2_FC values for differential abundance of transcripts and proteins between metformin and dimethylamine growth conditions are shown and coloured purple if log2FC > 1. Asterisks indicate significant adjusted *p*-values (adjp < 0.05). For each CDS, abundance of reads (RNA-Seq) and spectral counts (proteomics) in the metformin growth condition are both indicated as red circles with sizes proportional to their relative abundance compared to the genome average. The genome average per CDS of is of eight spectral counts for proteins and of 475 reads per CDS. The relative abundance of protein 4492 is at the genome average.

In this context, the 4488–4498 gene cluster appears particularly interesting with regard to metformin degradation (Figure 5). This cluster is conserved in synteny in all *Aminobacter* strains of known genome sequence and closely related to strain MD1 (protein identity > 80% for all CDS). Nevertheless, it features genes 4492 and 4498, two of the genes whose inactivation is associated with lack of growth with metformin in corresponding mutants. Notably, two independent mutants unable to grow with metformin were inactivated in gene 4498. Moreover, many genes of this cluster show significantly higher abundance during growth with metformin than with dimethylamine (Figure 5). This is in marked contrast with the pattern of most CDS with higher abundance during growth with metformin (see Table 2 and Figure 4). The two genes immediately downstream of gene 4492 and potentially co-transcribed with it are among the more abundant transcripts during growth with metformin in the entire genome (Figure 5, Table S5). They are annotated as agmatinases, an enzyme that hydrolyzes the guanidinium functional group of agmatine (the decarboxylation product of arginine) to putrescine. This is a strikingly similar reaction to the hydrolysis of metformin to guanylurea, as both reactions yield an amine and an urea as products. Thus, agmatinase homologs 4490 and 4491 represent attractive candidates for the enzyme catalyzing the growth-supporting transformation of metformin into guanylurea in strain MD1. The presence of genes encoding nickel incorporation proteins HypAB immediately upstream of agmatinase homologs 4490 and 4491 (Figure 5) is of particular interest in this context. Agmatinases are usually considered to be manganese-dependent enzymes [64], but nickel-dependent guanidine-degrading enzymes have recently been reported [65,66].

**Figure 6:**
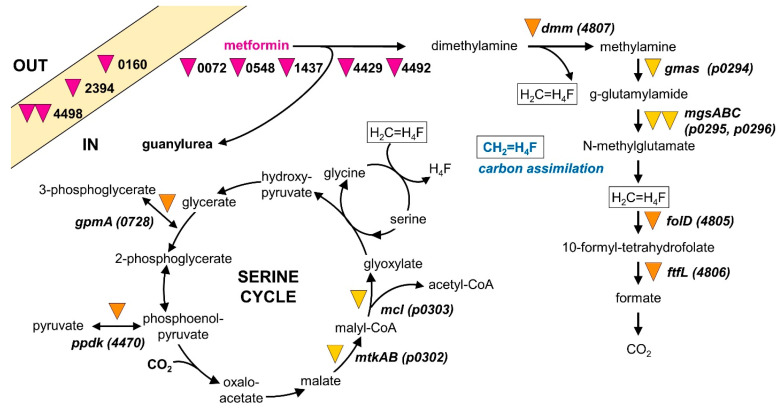
Metabolism of strain MD1 with metformin. Genes whose inactivation by mini-transposon insertion led to loss of growth with metformin are indicated by purple inverted triangles. Genes essential for growth with dimethylamine as well as with metformin are shown as orange or yellow inverted triangles to indicate their localization on the chromosome or plasmid paAMD1, respectively.

Taken together, our results from differential proteomics, transcriptomics, and mini-transposon mutagenesis suggest strong genes candidates for encoding the metformin hydrolase enzyme downstream of gene 4492, and also highlight the essential role of methylotrophic metabolic pathways in the growth of strain MD1 with metformin (Figure 6).

## 4. Discussion

Environmental studies have provided substantial evidence for the microbial transformation of metformin [16,20,23]. However, the detailed characterization of a strain capable of using metformin as a carbon and nitrogen source had yet to be reported. Our results showed that strain MD1 grows with metformin as the sole carbon, nitrogen and energy source. Strain MD1 obtains its energy from the oxidation of dimethylamine released by the hydrolysis of metformin, and releases guanylurea as an unmetabolized side-product into the medium. With guanylurea being the major metabolite of metformin detected in the environment [19,21,24], this pathway of metformin transformation may thus be quite widespread in metformin-degrading microorganisms.

Strain MD1 shows many characteristics suggestive of the recent adaptation to metformin as a nutrient source for growth. First, its genome sequence is very close to that of other *Aminobacter* strains which are unable to use metformin for growth (Figure 2, Figure S1, Supplementary Table S4). Second, patterns of gene expression and protein production for growth with metformin show very few differences from those for dimethylamine growth (Table 2). Third, almost all genes and gene clusters identified as essential for growth of strain MD1 with metformin (Table 3, Figure 6, Supplementary Table S7) are also found in closely related strains unable to grow with this compound. Finally, all such clusters are located on the chromosome of strain MD1 and not on plasmids, and they do not contain features suggesting horizontal gene transfer potential, such as IS elements or transposases, for example. Taken together, these observations support the notion that the capacity of strain MD1 to transform metformin for growth was developed recently.

Due to the large number of hydolases acting on C-N bonds predicted in its genome (Table 2), several experimental approaches were implemented to help identify molecular determinants of metformin utilisation in strain MD1. Functional genomics were used to detect transcripts and proteins more abundant during growth with metformin. In parallel, untargeted mutagenesis of the *Aminobacter* genome, followed by the screening of thousands of independent mutants, allowed to identify mutants that had lost the ability to grow with metformin. The number of essential genes (and associated gene clusters, considering the possibility of downstream polar effects resulting from gene inactivation by mini-transposon insertion) that were identified as essential for growth with metformin was quite large given that transformation from metformin to guanylurea represents a simple hydrolytic transformation (Figure 1). However, a requirement for several enzymes for hydrolysis of a guanidium functional group is not implausible. For example, the hydrolysis of guanylurea to guanidine was shown to proceed by carboxylation of the guanidine function followed by hydrolysis [25,26]. In addition, the enzymatic system for metformin transformation may also involve accessory proteins and cofactors, requiring additional genes for activity. In this study, a mutation that abolished growth with metformin was found in gene 4492 (Table 3, Figure 5) encoding a predicted nickel incorporation protein, suggesting that the enzymatic system involved in metformin degradation may be nickel-dependent.

The large number of gene clusters essential for metformin utilization may also reflect a requirement for essential processes other than the transformation of metformin itself, such as the transport of metformin, excretion of metabolites or regulation of metabolic fluxes. For example, gene 4498, essential for growth with metformin (Figure 5), encodes a nucleobase cation symporter homolog similar to the cytosine transport protein CodB, whose structure and proposed mechanism were recently described [67]. This CDS shows high differential abundance between metformin and dimethylamine conditions (Table S7, Figure 5), strongly suggesting that it may be involved in metformin import and/or guanylurea excretion. The relevance of two other transport genes inactivated in two other mutants unable to grow with metformin, i.e., gene 2394 coding for a DUF486 transporter, and gene 0160 for an ABC transporter component, are less clear. In addition, they were not detected by proteomics and their transcripts were not more abundant during growth with metformin (Table S7). As for the possibility of regulatory processes specifically associated with bacterial growth with metformin, they are tentatively suggested by the metformin-deficient mutant inactivated in gene 1437, annotated as a putative sensor domain-containing diguanylate cyclase.

A more detailed characterization of identified genes and gene clusters, including by heterologous expression, will allow for the definition of the precise requirements for the growth of strain MD1 with metformin—not only the required enzyme but also regarding metformin import, guanylurea export, and metabolic regulation. In addition, the essentiality of some genes identified by mini-transposon mutagenesis will have to be verified further. For example, gene 0548 defines the only gene cluster of strain MD1 identified as essential for growth with metformin that is not found in closely related *Aminobacter* strains with a known genome sequence. Its annotation as hydantoinase suggests a carbon-nitrogen amidohydrolase function. However, gene 0548 and its downstream gene 0549 showed only modest gene expression (Supplementary Table S5). In addition, corresponding proteins were not detected under any condition (Supplementary Table S6).This gene cluster therefore represents an unlikely candidate for the metformin hydrolase enzyme of strain MD1. Similarly, some other protein products of genes identified by mini-transposon mutagenesis as associated with growth with metformin were also undetected by proteomics (Table 3), including gene 0072 annotated as an amidohydrolase. It is possible that some secondary mutations were acquired during the isolation of some mutants, and this could account for their lack of growth with metformin. However, several lines of evidence speak against this being a significant issue of our mutagenesis experiment. First, many identified mutations were in genes for dimethylamine and methylamine utilization, demonstrating the adequacy of our mutagenesis and screening procedure. Similarly, two different mutants unable to grow with metformin were independently obtained with an independent insertion in transporter gene 4498, in the key genome region that also showed significantly higher gene expression and protein production during growth with metformin compared to growth with methylamine (Figure 5).

More generally, understanding the origin of the ability to grow with metformin in strain MD1 represents a very exciting perspective of our results for future work. Considering the low (ng/L to μg/L) concentrations of even heavily used pharmaceuticals such as metformin in wastewater treatment plants [2], it is debatable whether selective pressure for the growth-supporting use of metformin would operate in situations very different from those used in the present study, in which metformin concentrations were at least three orders of magnitude higher than in the environment. There is thus an intriguing possibility that strain MD1 was evolved in part during the enrichment process leading to its isolation. Further experiments with samples collected during enrichment may help to investigate this point in the future.

## 5. Conclusions

To our knowledge, strain MD1 is the first bacterium reported to grow with metformin as the sole carbon, nitrogen and energy source. Sequencing of its genome allowed us to assign it to the aerobic, facultatively methylotrophic species *Aminobacter niigataensis*, whose strain type is unable to grow with metformin, and to characterize metformin metabolism by functional genomics approaches. As demonstrated by LC/MS-MS, strain MD1 does not mineralize metformin, but rather grows with dimethylamine obtained by hydrolysis of metformin. This was confirmed by the generally similar patterns of higher transcript and protein abundance upon growth of strain MD1 with metformin and dimethylamine in comparison to growth with sucrose. Strikingly, most of the gene clusters identified by mutagenesis as essential for growth of strain MD1 with metformin are conserved in other *Aminobacter* strains unable to grow with this compound. This suggests the possibility of the recent evolution of the capacity to grow with metformin in strain MD1 following a limited number of mutations. We anticipate that our results will help to identify the proteins required for metformin metabolism in strain MD1, and to define bioindicator genes for monitoring metformin transformation in environments of interest, including the human microbiome.

## Figures and Tables

**Table 1:** Genome characteristics of *Aminobacter niigataensis* strain MD1.

Feature	Chromosome	Plasmid paAMD1	Plasmid pbAMD1	Plasmid pcAMD1
Coverage (-fold)	69.6	62.2	60.9	50.6
Topology	circular	circular	circular	circular
Size (kb)	4,947,010	363,670	24,942	21,575
GC (%)	63.5	63.3	62.9	59.6
rRNA operons	2	-	-	-
tRNA	50	-	-	-
CDS	5140	329	34	24
Coding density (%)	90.0	91.5	73.6	78.2
Completeness/contamination ^1^ (%)	99.3/0.4	NA ^2^	NA ^2^	NA ^2^
Repeat regions ^3^ (%)	4.8	0.9	24.0	0
CDS with EGGNOG annotation (%)	86.7	94.6	64.7	62.5
CDS with hydrolase prediction ^4^	599	59	3	3
CDS, specific hydrolase prediction ^5,6^	81	13	-	-
CDS, carboxylase prediction ^6^	42	2	-	-

^1^ as assessed by CheckM v1.0.11. ^2^ NA, not applicable. ^3^ as assessed by Nosferatu, MicroScope [44] platform. ^4^ Number of CDS with EGGNOG [56] or INTERPRO [57] annotation featuring the terms hydrolase or thiolase. ^5^ Number of CDS with EGGNOG [56] or INTERPRO [57] annotation featuring the terms carbon-nitrogen hydrolase, ureohydrolase, hydantoinase, amidohydrolase, or amidase. ^6^ Number of CDS with EGGNOG [56] or INTERPRO [57] annotation featuring the term.

**Table 2:** Overview of obtained comparative functional genomics data ^1^.

	Detected	M ≠ S ^2^	M *>* S ^3^	D ≠ S ^2^	D *>* S ^3^	M/D ≠ S ^2^	M/D > S ^4^	M ≠ D/S ^2^	M *>* D/S ^5^
RNA-Seq	5509	1189	113 (2.1)	1955	293 (5.3)	749	105 (1.9)	589	9 (0.2)
Proteomics	1581	125	52 (3.3)	194	58 (3.7)	83	39 (2.5)	22	6 (0.4)
Common	1580	90	43	158	52	70	37	16	6

^1^ Number of CDS are given, with % of total detected in brackets to facilitate comparison between RNA-Seq and proteomics experiments. ^2^ Differential abundance is based on adjusted *p*-value < 0.05. ^3^ Higher abundance is based on log_2_FC > 1 and adjusted *p*-value < 0.05. ^4^ log_2_FC > 1, adjusted *p*-value < 0.05 for both comparisons of growth with metformin (M) and dimethylamine (D) relative to sucrose (S). ^5^ log^2^FC > 1, adjusted *p*-value < 0.05 for both comparisons of growth with metformin (M) relative to dimethylamine (D) and to sucrose (S).

**Table 3:** Mini-transposon insertion loci associated with specific loss of growth with metformin.

			Gene	Transcriptomics			Proteomics		
		Conser-	Cluster	log_2_FC ^1^		Reads	log_2_FC ^1^		SC
CDS	Annotation	vation ^2^	Location	M vs. S	M vs. D	(M) ^3^	M vs. S	M vs. D	(M) ^4^
0072	amidohydrolase	(no) ^5^	2/2	0.12	0.10	279	- ^6^	-	ND ^7^
0160	ABC transporter	yes	22/31	−0.20	-	762	-	-	ND ^7^
0548	hydantoinase	no	1/2	0.15	0.37	1364	-	-	ND ^7^
1437	diguanylate cyclase	yes	2/2	−0.34	0.97	1020	-	-	ND ^7^
2394	transporter, DUF486	yes	1/3	−0.15	−0.51	308	-	-	ND ^7^
4429	thiolase	yes	1/4	0.03	0.03	1761	−0.06	0.58	5
4492	nickel incorporation	yes	2/23	0.10	**1.48**	19,236	−0.13	−0.03	1
4498	NCS transporter	yes	2/3	0.17	**1.78**	28,303	−0.91	**6.22**	9

^1^ Differential abundance in pairwise comparisons of metformin (M) with sucrose (S) and dimethylamine (D) growth conditions. Significant values of log2FC >1 are shown in bold. ^2^ Conserved in the 3 *Aminobacter niigataensis* strains MD1, MSH1 and type strain.^3^ Average CDS raw reads per replicate in the metformin condition (genome average 745 per CDS). ^4^ Average CDS spectral counts per replicate in the metformin condition (genome average 8). ^5^ Found in the *A. aganoensis* type strain, but not in the *A. niigataensis* type strain or strain MSH1. ^6^ not available. ^7^ not detected.

## Data Availability

See Section 2.8.

## Supplementary Materials

The following supporting information can be downloaded at: https://www.mdpi.com/article/10.3390/microorganisms10112302/s1. Figure S1: UV-VIS spectrometry analysis of metformin and guanylurea derivatized by the Sakaguchi reagent; Figure S2: Phylogenetic tree representation of genome clustering of selected *Aminobacter* strains; Supplementary Table S1: Biolog data for strain MD1 in comparison to other strains; Supplementary Table S2: Fatty acid composition data for strain MD1; Supplementary Table S3: Quantification of metformin and guanylurea by LC/MS-MS; Supplementary Table S4: DNA-DNA hybridisation analysis; Table S5: Comparative transcriptomics data; Supplementary Table S6: Comparative proteomics data; Supplementary Table S7: Mini-transposon insertion sites in metformin-negative mutants, and characteristics of the targeted genes.
