# Supplementary material for: A Methylotrophic Bacterium Growing with the Antidiabetic Drug Metformin as Its Sole Carbon, Nitrogen and Energy Source
Source: Microorganisms. 2022 Nov 21;10(11):2302. doi: 10.3390/microorganisms10112302 (PMC9699525; doi:10.3390/microorganisms10112302)
Supplement: Supplementary file 1 [file microorganisms-10-02302-s001.zip › chaignaud_supplementary_figures.pdf]

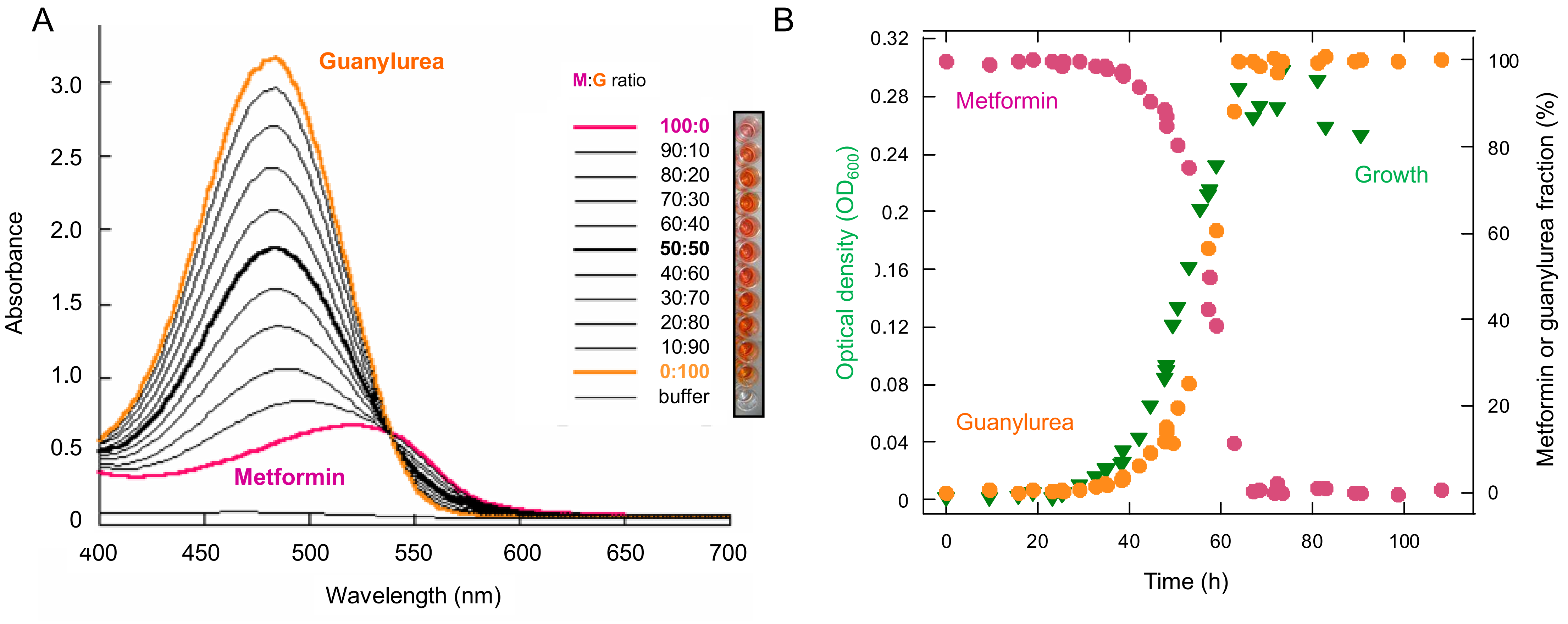

Figure S1. UV-VIS spectrometry analysis of metformin and guanylurea derivatized by the Sakaguchi reagent. **(A)** Spectra of samples of aqueous solutions of pure metformin (purple), guanylurea (orange), and mixtures of the two compounds in different ratios (2.5 mM total concentration of metformin and guanylurea) following derivatisation by the Sakaguchi reagent. **(B)** Representative growth curve of strain MD1 in mineral medium with 2.5 mM metformin as the sole carbon, nitrogen and energy source followed at 600 nm (green triangles). Metformin (purple circles) and guanylurea (orange squares) were estimated by colorimetric analysis with the Sakaguchi reagent, using reference compounds (2.5 mM in the same medium) analysed in parallel (see Materials and Methods).

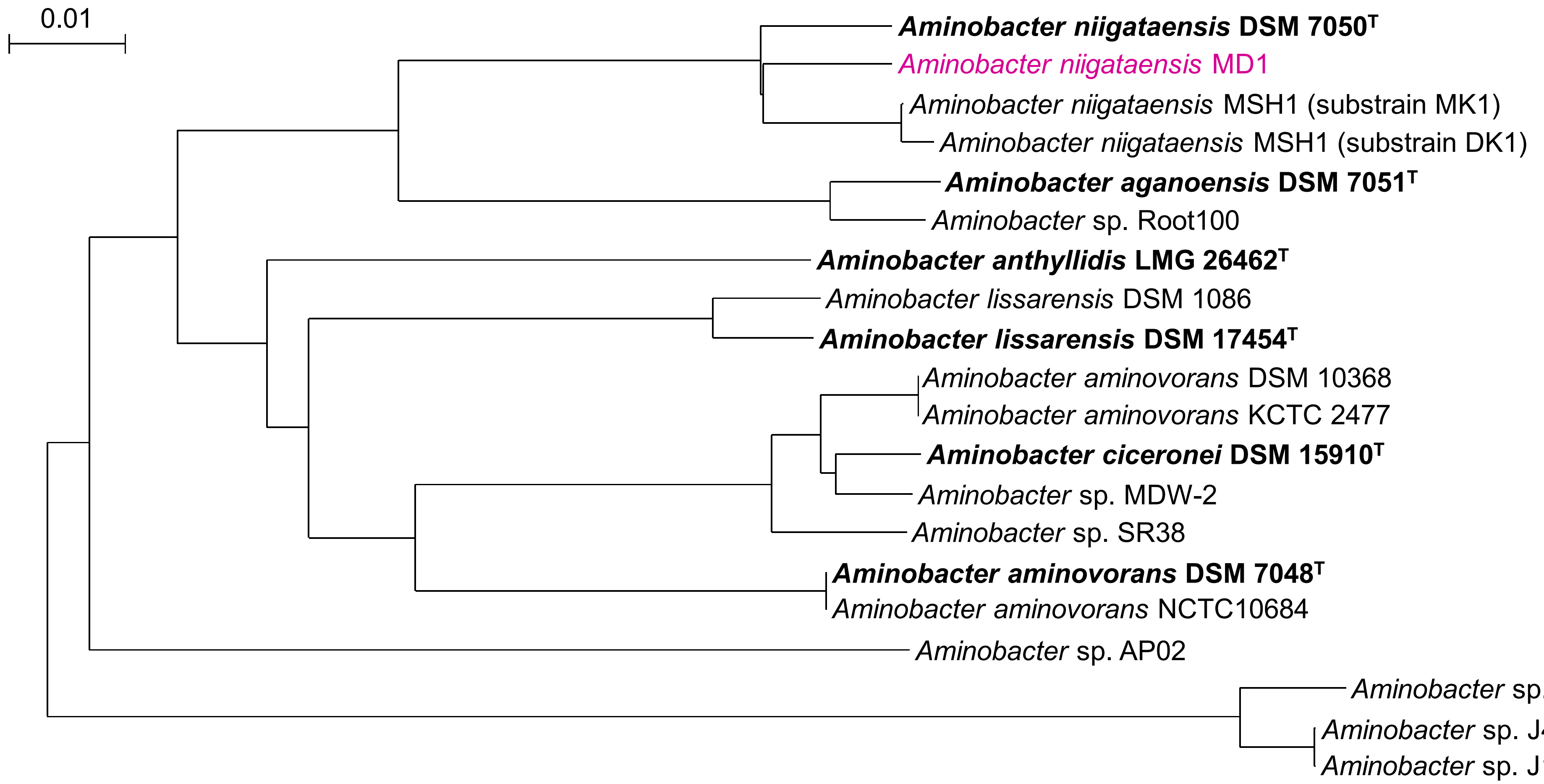

Figure S2. Similarity of metformin-degrading strain MD1 (purple) to other *Aminobacter* strains of known genome sequence evaluated by the genome clustering tool of MicroScope (https://microscope.readthedocs.io/en/3.16.0/content/compgenomics/genoclust.html). Type strains are shown in bold. Branch lengths are scaled as average nucleotide identity (ANI) distances.
